# Zerumbone Suppresses Enterotoxigenic *Bacteroides fragilis* Infection-Induced Colonic Inflammation through Inhibition of NF-κΒ

**DOI:** 10.3390/ijms20184560

**Published:** 2019-09-14

**Authors:** Soonjae Hwang, Minjeong Jo, Ju Eun Hong, Chan Oh Park, Chang Gun Lee, Miyong Yun, Ki-Jong Rhee

**Affiliations:** 1 Department of Biomedical Laboratory Science, College of Health Sciences, Yonsei University at Wonju, Wonju, Gangwon-do 26493, Koreaminjeongjo12@gmail.com (M.J.); raperm87@gmail.com (J.E.H.); cksdh9453@gmail.com (C.O.P.); dangsunsang@naver.com (C.G.L.); 2 Cell Therapy and Tissue Engineering Center, Yonsei University Wonju College of Medicine, Wonju, Gangwon-do 26426, Korea; 3 Department of Bioindustry and Bioresource Engineering, College of Life Sciences, Sejong University, Seoul 05006, Korea

**Keywords:** zerumbone, ETBF, BFT, inflammation, NF-κB

## Abstract

Enterotoxigenic *Bacteroides fragilis* (ETBF) is human intestinal commensal bacterium and a potent initiator of colitis through secretion of the metalloprotease *Bacteroides fragilis* toxin (BFT). BFT induces cleavage of E-cadherin in colon cells, which subsequently leads to NF-κB activation. Zerumbone is a key component of the *Zingiber zerumbet* (L.) Smith plant and can exhibit anti-bacterial and anti-inflammatory effects. However, whether zerumbone has anti-inflammatory effects in ETBF-induced colitis remains unknown. The aim of this study was to determine the anti-inflammatory effect of orally administered zerumbone in a murine model of ETBF infection. Wild-type C57BL/6 mice were infected with ETBF and orally administered zerumbone (30 or 60 mg/kg) once a day for 7 days. Treatment of ETBF-infected mice with zerumbone prevented weight loss and splenomegaly and reduced colonic inflammation with decreased macrophage infiltration. Zerumbone treatment significantly decreased expression of IL-17A, TNF-α, KC, and inducible nitric oxide synthase (iNOS) in colonic tissues of ETBF-infected mice. In addition, serum levels of KC and nitrite was also diminished. Zerumbone-treated ETBF-infected mice also showed decreased NF-κB signaling in the colon. HT29/C1 colonic epithelial cells treated with zerumbone suppressed BFT-induced NF-κB signaling and IL-8 secretion. However, BFT-mediated E-cadherin cleavage was unaffected. Furthermore, zerumbone did not affect ETBF colonization in mice. In conclusion, zerumbone decreased ETBF-induced colitis through inhibition of NF-κB signaling.

## 1. Introduction

Chronic inflammation is a major risk factor for many human diseases including cancer. Epidemiological studies suggest that at least 20% of all cancers are caused by chronic inflammatory conditions, such as the causative role of *Helicobacter pylori* and hepatitis B virus infections in gastric and liver cancer, respectively. Likewise, the chronic colitis exhibited by inflammatory bowel disease (IBD) patients is highly associated with colon cancer [1,2,3]. Although the underlying molecular mechanisms linking colitis and colon cancer are not clearly understood, gut microbes are thought to play a pivotal role in colitis-induced colon cancer progression. *Bacteroides fragilis* is a gram-negative, obligate anaerobe that is found consistently, but in low numbers, in the gut microbial community of humans [4]. Molecules elaborated by *B. fragilis* shape and limit inflammation to the mutual benefit of host and bacterium [5]. However, the effects on host health are highly strain dependent. Enterotoxigenic *B. fragilis* (ETBF) is a particular subtype of *B. fragilis* characterized by production of the secreted *B. fragilis* toxin (BFT). ETBF is a causative agent of acute diarrhea among humans and livestock [6,7,8] and is correlated with active inflammatory status in patients with IBD [9]. The overrepresentation of ETBF strains are detected in the microbiome of colorectal cancer (CRC) patients [10], and their physical association with neoplastic tissue further implicates these organisms in human disease [11]. ETBF virulence has been attributed to the activity of BFT [12] which enhances colon tumorigenesis and exacerbates IBD-like symptoms in mouse models [13,14]. *B. fragilis* is also the leading cause of anaerobic sepsis [15] in which BFT is required for pathogenesis [16]. BFT, a 20 kDa zinc metalloprotease, severely alters cell-to-cell adherens junctions in the colon epithelium through ectodomain cleavage of E-cadherin [17], disrupting barrier function and activating the NF-κB signal pathway to induce pro-inflammatory genes [18,19,20], such as IL-8. Moreover, a myriad of pro-inflammatory cytokines is subsequently produced, most notably the Th17 cytokine IL-17A, required for ETBF colitis-promoted tumorigenesis [21]. Currently, no therapy has proven efficacious in reducing disease burden of ETBF, and controversy persists as to whether treatment with antibiotics is helpful [22,23,24]. A previous in vivo study showed that treatment of cefoxitin, a second-generation cephamycin antibiotic, cleared ETBF colonization in C57BL/6 mice, thereby reducing ETBF-induced inflammatory response in the colons of mice [25]. However, antibiotics, even when used for short periods of time, may raise the issues of both toxicity and the emergence of bacterial antibiotic resistance [26,27]. In addition, the use of antibiotics heavily disrupts the homeostasis of the gut microbiome, thereby inducing dysbiosis of gut microbiome [4,28]. Dysbiosis may hamper vital normal physiologic functions such as nutrient supply, vitamin production, and protection from pathogens [29]. It is thus an enticing prospect that natural products may disrupt the cycle of ETBF-induced pathogenic inflammation thereby lessening the disease burden in place of conventional antibiotics.

Plant extracts containing various polyphenols have been shown to reduce inflammation with less accompanying toxicity compared to synthetic antibiotics [30]. Zerumbone, a naturally occurring phytochemical and an extract of *Zingiber zerumbet*, is reported to possess many pharmacologic properties [31,32,33,34] and is widely used in herbal medicine. Moreover, zerumbone has proven to exhibit marked anti-carcinogenic, anti-inflammatory, and antioxidant properties [35,36,37]. A recent study showed that zerumbone significantly inhibited biofilm formation and eradicated established biofilms caused by ETBF [38]. Thus, zerumbone may be a potential agent for controlling inflammation associated with ETBF infection, thereby preventing ETBF-mediated tumorigenesis. The aim of the present study is to evaluate the anti-inflammatory effect of zerumbone using the murine experimental model of ETBF infection. We found that zerumbone reduces colon inflammation in mice infected with ETBF. However, protective effect of zerumbone was independent of ETBF colonization or E-cadherin cleavage by BFT but rather appeared to be mediated by inhibition of BFT-induced NF-κB signaling in colon epithelial cells. It is anticipated that our study will contribute to the growing evidence on the beneficial role of zerumbone in bacteria-mediated colitis.

## 2. Results

### 2.1. Zerumbone Reduced Indirect Parameters of ETBF-Induced Colonic Inflammation in Mice

**Figure 1:**
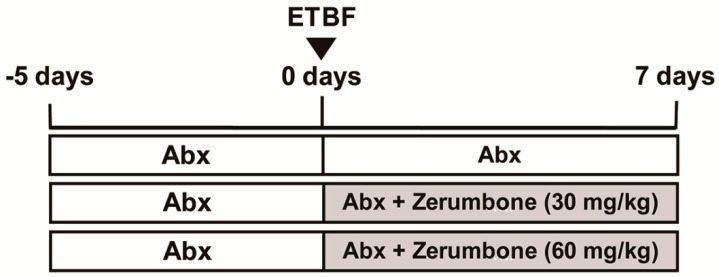
Experimental design. C57BL/6 female mice were provided water ad libitum containing clindamycin/gentamicin for 5 days before Enterotoxigenic *B. fragilis* (ETBF) infection. Wild-type ETBF was orally inoculated, and the antibiotic cocktail continued for an additional 7 days. During ETBF infection, C57BL/6 mice were administered with zerumbone (30 or 60 mg/kg, p.o.,) daily. Total experimental period was 12 days. C57BL/6 mice were sacrificed at day 7 post-infection.

To assess the anti-inflammatory effects of zerumbone on ETBF-induced colon inflammation, C57BL/6 mice were provided with drinking water containing clindamycin and gentamicin 5 days prior to wild-type ETBF (WT-ETBF) oral inoculation (Figure 1). WT-ETBF (1 × 10^9^ colony-forming units (CFU)) were orally inoculated once on day 0 (Figure 1). After WT-ETBF infection, C57BL/6 mice were orally administered zerumbone (30 or 60 mg/kg) once daily. Antibiotic treatment was continued for the duration of the experiment. Mice were sacrificed after one week post-infection. During the WT-ETBF infection period, freshly collected stool pellets were cultured to assess colonization of WT-ETBF. WT-ETBF colonization was comparable in mice infected with WT-ETBF alone and WT-ETBF-infected mice administered daily with zerumbone (60 mg/kg) (9.1 × 10^8^ and 1.6 × 10^8^ CFU per gram of stool, respectively) at day 3 and remained constant during the experimental period (Supplementary Figure S1), suggesting that treatment of ETBF infection with daily zerumbone (30 or 60 mg/kg) for 1 week by oral gavage did not affect ETBF colonization (Supplementary Figure S1).

**Figure 2:**
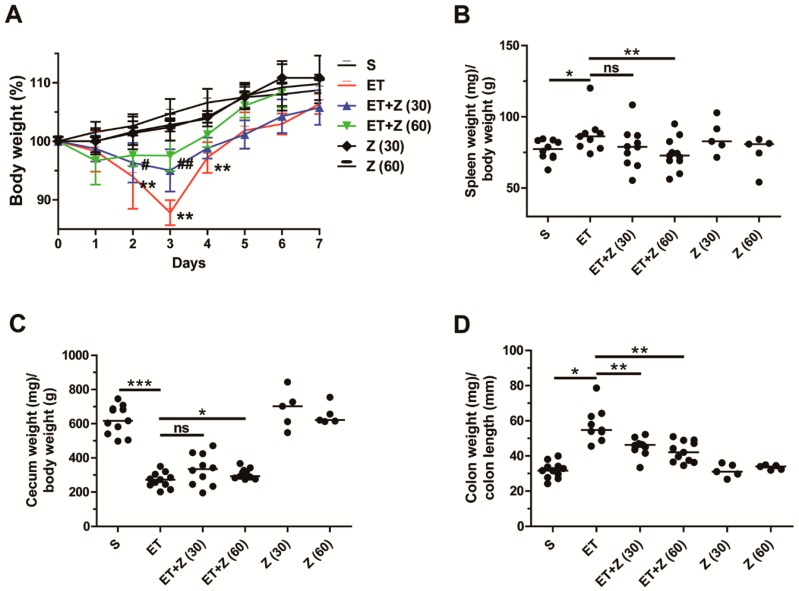
Clinicopathologic analysis of ETBF-infected mice administered with zerumbone. C57BL/6 female mice were infected with WT-ETBF. During ETBF infection, the zerumbone-treated group was orally gavaged with zerumbone (30, 60 mg/kg, p.o., once a day). (**A**) Body weight. The daily body weight of individual mice was normalized to the starting body weight (%). ET + Z (30) vs. ET, ^#^ *p* < 0.05, ^##^ *p* < 0.01; ET + Z (60) vs. ET, * *p* < 0.05, ** *p* < 0.01; significances between treated groups were determined using Mann–Whitney *U* test. (**B**) Spleen weight (mg)/body weight (g). (**C**) Cecum weight (mg)/body weight (g). (**D**) Colon weight (mg)/colon length (mm). Colon weight (mg)/colon length (mm) ratio were measured at day 7 post-infection. S, sham; ET, ETBF; Z (30), Zerumbone (30 mg/kg); Z (60), zerumbone (60 mg/kg). Scatter plot. Horizontal bar, median. * *p* < 0.05, ** *p* < 0.01, *** *p* < 0.001. ns, no statistical significance.

It has been shown that 2 to 3 days after ETBF colonization, C57BL/6 mice exhibit body weight loss as a result of colon inflammation [12]. ETBF-infected C57BL/6 mice are characterized by reduced cecum weight and increased splenomegaly, all indirect indicators of colonic inflammation [12]. Furthermore, the extent of colon inflammation positively correlates with increased colon weight/colon length ratio [16]. We therefore examined spleen weight, cecum weight, and colon weight/colon length ratio after 7 days post-infection. Body weight was measured daily. We found that, as expected, WT-ETBF-infected mice showed the greatest decrease in body weight at day 3 post-infection (Figure 2A). In contrast, ETBF-infected mice administered zerumbone at either 30 mg/kg or 60 mg/kg showed a significantly less decrease in body weight at day 3. The increased spleen weight observed in ETBF-infected mice also decreased in zerumbone-treated ETBF-infected mice but reached statistical significance at only zerumbone used at 60 mg/kg (Figure 2B). Likewise, the decreased cecum weight observed in ETBF-infected mice also slightly increased upon administration with zerumbone (60 mg/kg) (Figure 2C). Lastly, the increased colon weight/colon length ratio observed in ETBF-infected mice was significantly decreased in ETBF-infected mice treated with zerumbone (30 or 60 mg/kg) (Figure 2D). Taken together, zerumbone treatment (60 mg/kg) was effective in decreasing all indirect parameters of colonic inflammation observed in ETBF-infected mice. Zerumbone treatment alone did not alter any of the parameters assessed compared to the sham control (Figure 2A–D).

### 2.2. Zerumbone Decreased ETBF-Induced Histologic Damage in Mouse Colon

**Figure 3:**
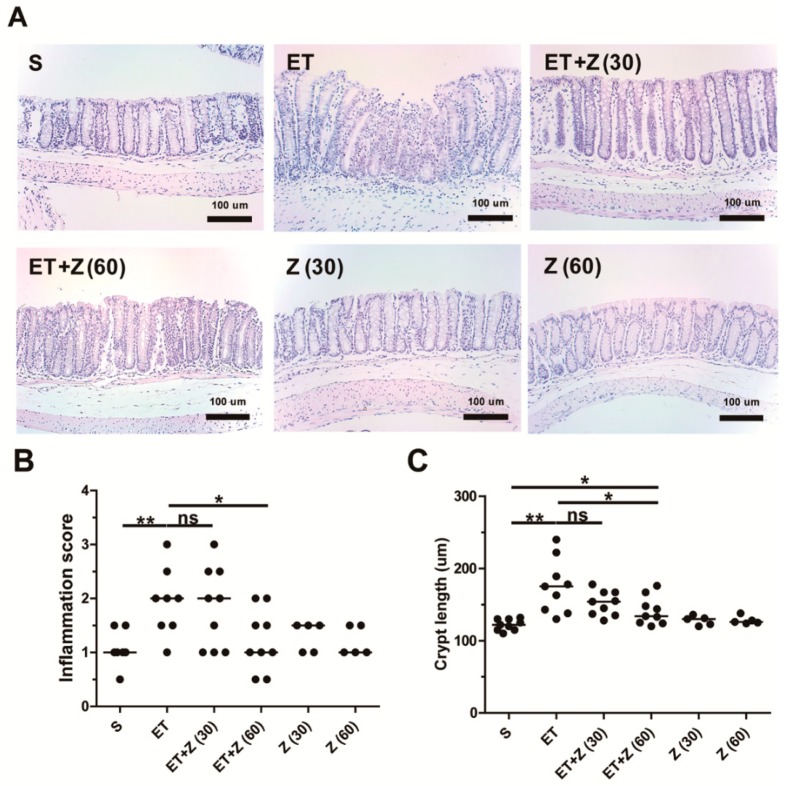
Histopathology analysis of distal colon in ETBF-infected mice. Formalin-fixed paraffin-embedded (FFPE) distal colonic tissues obtained from C57BL/6 mice at day 7 post-infection were stained with hematoxylin and eosin (HE). Representative images are shown. Histologic scores were evaluated using HE slides. S, sham; ET, ETBF; Z (30), Zerumbone (30 mg/kg); Z (60), Zerumbone (60 mg/kg). (**A**) Histology of distal colon, ×200 magnification; Scale bar, 100 μm, (**B**) Inflammation score. (**C**) Crypt length. Scatter plot. Horizontal bar, median. * *p* < 0.05, ** *p* < 0.01. ns, no statistical significance.

ETBF-induced colitis in mice involves colonic infiltration of inflammatory cells, colonic epithelial cell exfoliation, and colonic crypt elongation as a result of BFT-induced destruction of the epithelial barrier [12]. To determine if zerumbone decreases ETBF-induced inflammation, hematoxylin and eosin (HE) staining was performed on the colon and the inflammation score examined. Histologic staining showed characteristic epithelial cell rounding and subsequent exfoliation, inflammatory cell infiltration, and increased crypt length (Figure 3A). In contrast, ETBF-infected mice administered zerumbone showed a decrease in inflammation, with higher dose of zerumbone (60 mg/kg) showing a more protective effect. Collectively, the inflammation score and crypt length all indicate that ETBF-infected mice administered 60 mg/kg zerumbone decreased these parameters, although 30 mg/kg zerumbone was also somewhat protective but did not reach statistical significance (Figure 3B,C).

### 2.3. Zerumbone Decreased Infiltration of Macrophage in Distal Colon of ETBF-Infected Mice

**Figure 4:**
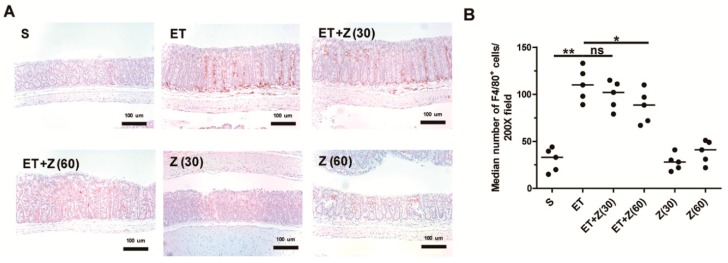
Histological analysis of macrophage infiltration in distal colon of ETBF-infected mice. FFPE distal colonic tissues obtained from mice at day 7 post-infection were stained with anti-F4/80 antibody and counterstained with hematoxylin. Representative images are shown. S, sham; ET, ETBF; Z (30), Zerumbone (30 mg/kg); Z (60), Zerumbone (60 mg/kg). (**A**) Immunohistochemistry (IHC) for F4/80^+^ cells, ×200 magnification; Scale bar, 100 μm. (**B**) Median number of F4/80^+^ cells at 200× field. Scatter plot. Horizontal bar, median. * *p* < 0.05, ** *p* < 0.01. ns, no statistical significance.

Both clinical and non-clinical studies have demonstrated that inflammation and the accompanying tissue infiltrating macrophages are conducive to progression of chronic inflammation [39,40]. The Sears group also showed that ETBF-infected APC^Min/+^ mice exhibited increased infiltration of macrophages [41,42]. Having observed that zerumbone treatment decreased ETBF infection-induced histologic inflammation in mice, we hypothesized that zerumbone decreased macrophage infiltration in mice colons. To test this hypothesis, distal colon tissues were examined for infiltrating macrophages via immunohistochemistry (IHC). IHC analysis showed that ETBF infection increased infiltrated macrophage in the distal colon, whereas ETBF-infected mice administered zerumbone (60 mg/kg) significantly down-regulated macrophage infiltration (Figure 4A,B).

### 2.4. Zerumbone Reduced Expression of Pro-Inflammatory Cytokines and NF-κB Pathway Related Proteins in Colon of ETBF-Infected Mice

**Figure 5:**
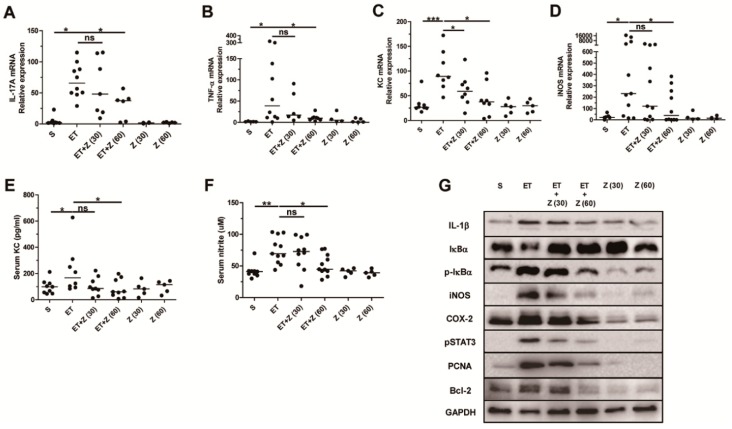
Analysis of expression of pro-inflammatory genes and proteins in distal colon of ETBF-infected mice. Distal colon was analyzed for mRNA expression of IL-17A, TNF-α, KC, and iNOS by RT-PCR. Serum KC level was examined by ELISA. Serum nitrite level was examined by nitric oxide assay. S, sham; ET, ETBF; Z (30), Zerumbone (30 mg/kg); Z (60), Zerumbone (60 mg/kg). (**A**) IL-17A expression. (**B**) TNF-α expression. (**C**) KC expression. (**D**) iNOS expression. (**E**) Serum KC. (**F**) Serum nitrite. (**G**) Western blot analysis of lysate in distal colon. Scatter plot. Each dot represents one mouse. Horizontal bar, median. * *p* < 0.05, ** *p* < 0.01, *** *p* < 0.001. ns, no statistical significance.

Bacterial infection-induced colitis is mediated by inflammatory mediators, such as cytokines and reactive oxygen species [43]. ETBF infection is characterized by induction of the pro-inflammatory IL-17A cytokine in colon of C57BL/6 mice [14]. IL-17A-mediated inflammation is augmented by IL-1β and/or TNF-α cytokines in vitro and in vivo [44]. In addition, IL-17A or TNF- α cytokine induces IL-8 (a human homologue of KC) expression in human colon adenocarcinoma cells [42]. Among the reactive oxygen species, nitric oxide produced by inducible nitric oxide synthase (iNOS) mediates the immune host defense in bacterial infections in order to eliminate bacteria in tissues [45]. In order to examine whether zerumbone decreases the induction of inflammatory cytokines and reactive oxygen species, colon tissues from ETBF-infected mice administered zerumbone were analyzed for expression of IL-17A, TNF-α, KC, and iNOS expression by real-time PCR. In addition, serum KC and nitrite levels were examined by ELISA and nitric oxide assay, respectively. Results indicate that ETBF-infected mice administered zerumbone (60 mg/kg) showed a decrease in IL-17A, TNF-α, KC, and iNOS expression compared with ETBF-infected mice (Figure 5A–D). KC and nitrite (a metabolite of nitric oxide) levels in the serum were also decreased in ETBF-infected mice administered zerumbone (60 mg/kg) consistent with data for the iNOS and KC expression data (Figure 5E,F).

Induction of IL-17A, TNF-α, KC and iNOS are mediated by NF-κB activation [46]. NF-κB controls not only expression of the inflammatory genes (IL-1, iNOS, COX-2 and p-STAT3) but also promotes cell proliferation and induction of survival proteins (PCNA and Bcl-2) [47,48]. In order to confirm and expand upon the mRNA expression data, we further analyzed protein levels of other inflammatory cytokines, proteins of the NF-κB signaling pathway, proliferation proteins and cell survival proteins by Western blot. We found that in the colonic tissues, ETBF-infected mice exhibited decreased IκBα and increased p-IκBα (an indicator of activated NF-κB pathway) compared to non-infected sham mice (Figure 5G). In contrast, ETBF-infected mice administered zerumbone (60 mg/kg) showed increased IκBα and decreased p-IκBα (Figure 5G). Moreover, the inflammatory proteins (IL-1β, COX-2 and p-STAT3), proliferative protein PCNA and survival protein Bcl-2 were increased in colons of ETBF-infected mice compared to sham mice, while the inflammatory, proliferative, and survival indicators were decreased in ETBF-infected mice treated with zerumbone (60 mg/kg) compared to mice infected with ETBF alone (Figure 5G). COX-2 was also decreased in colons of mice given only zerumbone (30 or 60 mg/kg) compared to sham group (Figure 5G), suggesting that oral intake of zerumbone may decrease basal levels of COX-2 in colons of mice. Collectively, these data support the hypothesis that zerumbone treatment decreases ETBF infection-induced colon inflammation.

### 2.5. Zerumbone Inhibited NF-κB Signaling and IL-8 Expression, but not Cleavage of E-Cadherin, by BFT in HT29/C1 Cells

**Figure 6:**
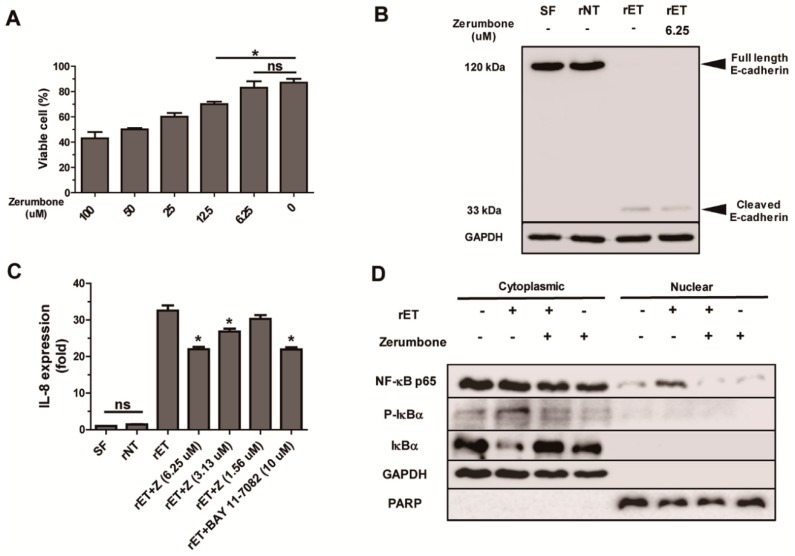
Zerumbone does not inhibit BFT-induced E-cadherin cleavage but inhibits NF-κB. HT29/C1 cells were treated with zerumbone with or without rETBF culture supernatants (1:10). (**A**) Cell viability of HT29/C1 cells treated with zerumbone (6.25 to 100 μM) for 24 h. (**B**) Western blot of E-cadherin in HT29/C1 cultured with rETBF culture supernatant and zerumbone (1.56 to 6.25 μM) for 1 h. (**C**) Real time-PCR analysis of IL-8 expression in HT29/C1 cells treated with rETBF culture supernatant and zerumbone (1.56 to 6.25 μM) or Bay11-7082 (a chemical NF-κB inhibitor) for 3 h. Data are expressed as the mean ± SEM from three independent experiments. (**D**) Western blot of NF-κB p65, p-IκBα, IκBα, GAPDH, and PCNA in nuclear fraction and cytoplasmic fraction of HT29/C1 cells treated with rETBF culture supernatant with or without zerumbone (6.25 μM) for 3 h. GAPDH was used as an internal control for cytosolic fraction and PCNA was used as an internal control for nuclear fraction.

BFT treatment of human colonic carcinoma cell line HT29/C1 leads to cleavage of E-cadherin (a component of adherence junction), yielding a 33 kDa cytoplasmic E-cadherin fragment [12,17]. The cleavage of E-cadherin in turn induces NF-κB signaling and IL-8 expression [20]. To determine if the protective effects exerted by zerumbone in mice was due to inhibition of E-cadherin cleavage, BFT-treated HT29/C1 cells were cultured with zerumbone and E-cadherin cleavage examined in vitro. First, to determine the highest concentration of zerumbone that does not induce cytotoxicity, HT29/C1 cells were cultured with various concentrations of zerumbone (6.25 to 100 μM) for 24 h and cell viability determined by trypan blue exclusion assay. Results show that cell viability of HT29/C1 cells appears to decrease from 12.5 μM and higher concentration of zerumbone (Figure 6A). Therefore, we used 6.25 μM of zerumbone to evaluate the effects of zerumbone on BFT-induced E-cadherin cleavage, NF-κB signaling, and IL-8 expression in vitro.

HT29/C1 cells were treated with rETBF culture supernatant (rET; culture media of *B. fragilis* secreting active BFT) in the presence of zerumbone. Additionally, HT29/C1 cells were treated with rNTBF culture supernatant (rNT; culture media of *B. fragilis* secreting catalytically inactive BFT) in order to confirm contribution of enzymatic activity to BFT-induced cellular alterations. After 1 h, Western blot was performed to detect E-cadherin cleavage fragments in HT29/C1 lysates (Figure 6B). HT29/C1 cells treated with rETBF culture supernatant and zerumbone (6.25 μM) showed no full-length 120 kDa E-cadherin similar to HT29/C1 cells treated with rETBF culture supernatant (Figure 6B). These results indicate that zerumbone does not inhibit E-cadherin cleavage. We next examined the impact of zerumbone on BFT-induced IL-8 expression in HT29/C1 cells (Figure 6C). HT29/C1 cells were treated with zerumbone (1.56 to 6.25 μM) and rETBF culture supernatant for 3 h. We found that BFT-treated HT29/C1 cells cultured with 6.25 μM of zerumbone showed the highest decrease in IL-8 expression (Figure 6C). Treatment with 3.13 μM zerumbone was less effective, and 1.56 μM zerumbone showed no protective effect in inhibiting IL-8 expression (Figure 6C). The addition of a NF-κB inhibitor (BAY 11-7082; 10 μM) to BFT-treated HT29/C1 cells reduced IL-8 expression to comparable levels to zerumbone treatment.

NF-κB activation involves the phosphorylation of IκBs, which is followed by IκBs degradation and the subsequent migration of NF-κB p65 from the cytoplasm to the nucleus [47]. Therefore, we investigated whether zerumbone decreased BFT-induced NF-κB signaling in HT29/C1 cells. HT29/C1 cells were treated with rETBF culture supernatant and zerumbone (6.25 μM) for 3 h. After 3 h, nuclear lysates of HT29/C1 cells were extracted via nuclear/cytoplasm fractionation kit, followed by Western blot analysis to detect nuclear or cytoplasmic p-IκBα, IκBα, and NF-κB p65. Results indicate that HT29/C1 cells co-treated with zerumbone (6.25 μM) and rETBF culture supernatant showed reduced cytoplasmic p-IκBα and increased nuclear NF-κB p65 and increased cytoplasmic IκBα compared to HT29/C1 cells treated with rETBF culture supernatant alone (Figure 6D). These results suggest that zerumbone treatment decreases BFT-induced IL-8 expression through inhibition of NF-κB signaling irrespective of E-cadherin cleavage.

## 3. Discussion

To our knowledge, the results presented here are the first to show anti-inflammatory effects of zerumbone in ETBF infection-induced colitis. Histologic analysis showed decreased colitis in zerumbone (60 mg/kg)-treated ETBF-infected mice compared with ETBF-infected. Moreover, inflammation-associated cytokines and proteins were decreased in the colon of zerumbone (60 mg/kg)-treated mice. Zerumbone-treated HT29/C1 cells exhibited inhibition of NF-κB signaling and IL-8 expression by BFT. Our in vivo and in vitro zerumbone data implicate inhibition of NF-κB signaling but not E-cadherin cleavage as crucial to the protective effects of zerumbone in ETBF-mediated colitis. A previous study showed that ETBF colonization causes acute colon inflammation in C57BL/6 mice through active BFT secretion [12]. BFT-induced colonic inflammation absolutely requires initiation of E-cadherin cleavage and subsequent induction of NF-κB signaling [17,19,42]. As far as we know, BFT does not directly activate dendritic cells and TLR-mediated pathway in vitro [12]. However, zerumbone treatment does not inhibit BFT-induced E-cadherin cleavage in colonic epithelial cells (Figure 5B), suggesting that the protective effect of zerumbone in ETBF-mediated colitis in mice is not due to inhibition of E-cadherin cleavage.

ETBF-induced colonic inflammation promotes IL-17A cytokine-promoted tumorigenesis in APC^Min−/+^ mice [14]. During ETBF-infection, IL-17A is produced by γδ T cells and Th17 cells [21]. Moreover, ETBF infection-mediated tumorigenesis is contributed by STAT3 signaling of Th17 cells or colon epithelial cells in APC^Min−/+^ mice [42]. In the current study, we found that zerumbone decreases pSTAT3 and IL-17A in the colon of ETBF-infected mice, but we cannot discern whether the decrease in colonic IL-17A expression by zerumbone is due to decreases in IL-17A producing γδ T cells and/or Th17 cells. As NF-κB also induces IL-17A expression [49], it is possible that zerumbone may not affect γδ T cells and/or Th17 numbers but rather the NF-κB pro-inflammatory pathway. The activation of the NF-κB pathway in turn may stimulate key chemokines, including KC (murine functional homologue of human IL-8), inducing macrophage recruitment in a CXCR2-dependent fashion [42]. Consistent with in vitro data showing decrease in IL-8 expression in vitro, zerumbone also decreased macrophage in distal colon of mice with decreased serum KC, a functional homolog of human IL-8. Although infiltrating macrophage mediates bacterial clearance in inflamed tissues [50,51], chronic inflammation mediated by macrophage might disrupt intestinal homeostasis through production of reactive oxygen species to kill bacteria in tissues [52], which might promote gut fibrosis and polyp formation.

A previous in vivo study showed that treatment of cefoxitin cleared ETBF colonization in C57BL/6 mice, thereby reducing ETBF-induced inflammatory response in distal colons of mice [25]. However, antibiotics, even used for short periods may raise the issue of both toxicity and the emergence of bacterial antibiotic resistance. The number of reports of multidrug-resistant *B. fragilis* strains has increased in the past decade [53,54,55]. In particular, at NYU Langone Medical Center, resistance rates for 361 *Bacteroides* isolates were evaluated over a 5-year time period which demonstrated overall resistance rates of 5% (17/361) to metronidazole, 4% (13/361) to carbapenems, and 0.3% (1/361) to both carbapenems and metronidazole [56]. In addition to this, the use of antibiotics heavily disrupts the ecology of the human microbiome [57]. Therefore, it is important to find natural products to reduce inflammation induced by ETBF infection as natural products were reported to decrease not only inflammation in tissues but also intestinal dysbiosis. We are currently investigating gut microbiome alterations in ETBF-infected mice given zerumbone. In the current study, oral administration of zerumbone did not affect ETBF colonization. Furthermore, zerumbone administration alone showed no adverse effects in mice.

In the canonical pathway, NF-κB is bound to IκB and thus inactive. Phosphorylation of IκB by IKK results in ubiquitination and subsequent degradation of IκB. The NF-κB becomes dissociated from IκB and in turn translocates into the nucleus to activate transcription of a myriad of genes. Using the CDOCKER program, which models putative binding of proteins with ligands, Fatima et al. suggested that that zerumbone binds to IKKβ thus inhibiting degradation of IκB and dissociation from NF-κB [58]. This hypothesis provides a mechanistic explanation as to how zerumbone suppresses ETBF-induced NF-κB activation in vitro. Furthermore, this putative molecular mechanism may explain the anti-inflammatory effects of zerumbone in ETBF-infected mice. However, it is clear that the anti-inflammatory effects of zerumbone in our mouse experiments are down-stream of E-cadherin cleavage as zerumbone did not inhibit BFT-induced E-cadherin cleavage. A recent study showed that human colon mucosal biofilms obtained from tumor hosts or healthy individuals are required for tumorigenesis in murine models of APC^Min−/+^ mice [59]. In clinical studies, colonic bacterial biofilm obtained from patients with CRC harbored inflammatory gut microbes, such as ETBF, polyketide synthase (Pks)^+^ *E. coli*, and/or *Fusobacterium nucleatum* [11,60,61]. These bacteria abundant in the gut biofilm might promote colon inflammation [62]. Recently, Kim et al. reported that zerumbone suppressed biofilm formation by microbes including ETBF in vitro [38,63]. In that study, zerumbone directly inhibited *B. fragilis* growth in vitro at a MIC of 32–48 μg/mL. The anti-microbial activity of zerumbone in the mouse intestine against ETBF was not observed in our study emphasizing the incongruent results of in vitro versus in vivo studies. It is still possible that zerumbone inhibited biofilm formation in ETBF-infected mice, which provided another layer of protective effect in addition to inhibition of the NF-κB pathway. Thus, the protective effect of zerumbone in ETBF-infected mice is likely multi-faceted.

## 4. Materials and Methods

### 4.1. Mice

Female C57BL/6 mice, 8 weeks of age, were bred in-house from mice originally purchased from Raon-Bio company (Yongin, Republic of Korea) and maintained under specific pathogen-free conditions. C57BL/6 mice were housed in cages, fed ad libitum, and maintained at 25 °C with a 12 h light/dark phase cycle. All animal housing and experimental procedures have been reviewed and approved by the Institutional Animal Care and Use Committee of Yonsei University at Wonju (YWCI-201901-002-01) and Institutional Biosafety Committee of Yonsei University at Wonju (201809-P-005-01). All experiments were performed to conform to relevant guidelines and regulations under the Institutional Animal Care and Use Committee of Yonsei University at Wonju and Institutional Biosafety Committee of Yonsei University at Wonju. Sample size estimates for animal experiments were based on prior animal modeling studies utilized within the laboratory for investigation of ETBF infection and colon inflammation. At the time of weaning, C57BL/6 mice were randomly distributed for use in experimentation.

### 4.2. Cell Culture

The human colon adenocarcinoma cell line HT29/C1 was grown to 80% confluency in 6-well culture plates in Dulbecco′s modified Eagle medium (DMEM, 4.5 g/L glucose, l-glutamine) supplemented with 10% fetal bovine serum (FBS) and penicillin (100 U/mL)/streptomycin (100 μg/mL). HT29/C1 cells were grown at 37 °C in a cell culture incubator with 10% CO_2_. For in vitro experiments, filter-sterilized (0.45 μm) bacterial culture supernatants of *B. fragilis* recombinant strains 9343 (rETBF) (pFD340::P-*bft*, secretes wild-type BFT-2) and 9343 (rNTBF) (pFD340::P-*bft*ΔH352Y, secretes mutant biologically inactive BFT owing to a single nucleotide point mutation in the BFT-2 metalloprotease domain) were used for investigating effects of zerumbone in BFT-treated HT29/C1 cells. Purified zerumbone was purchased from Sigma-Aldrich. Adherent HT29/C1 cells were washed twice with PBS before treatment with culture supernatant of each *B. fragilis* recombinant strain at specified concentrations in serum-free DMEM in order to prevent BFT neutralization by serum proteins. HT29/C1 cells were incubated with the NF-κB inhibitor (BAY 11-7085; Calbiochem, San Diego, CA, USA) for 20 min before the addition of culture supernatant from *B. fragilis* recombinant strain and then continuously during the experiment unless otherwise described. All culture media and reagents were purchased from GIBCO Life Technologies (Rockville, MD, USA) unless otherwise stated.

### 4.3. ETBF Infection in Mice and Zerumbone Treatment

The wild-type ETBF strain secreting BFT-2 (*B. fragilis* 86-5443-2-2) was used for inducing colitis in C57BL/6 mice. The wild-type *Bacteroides* strain used in this study is resistant to clindamycin and gentamicin. All bacterial strains were a generous gift from Cynthia Sears and Augusto Franco-Mora (Johns Hopkins University, Baltimore, MD, USA). C57BL/6 mice were administered gentamicin (300 mg/L) and clindamycin (100 mg/L) in the drinking water for five days prior to and throughout the course of bacterial infection. For preparation of bacterial cultures for oral inoculation, overnight cultures of *B. fragilis* were subcultured at a 1:50 ratio into fresh brain heart infusion broth (BHIB) and grown for 48 h. Bacteria from 50 mL culture were centrifuged at 12,000× *g* for 20 min. Thereafter, bacteria were resuspended in 1.0 mL phosphate-buffered saline (PBS) to yield a concentration of 10^9^ colony-forming units (CFU)/mL. Bacterial infection was performed by oral gavage of 200 μL of each inoculum. The CFU in each inoculum were confirmed by serial dilution plating on BHIB agar. To determine bacterial colonization in mice, fresh fecal pellets were collected from mice, weighed, and vortexed in 1 mL PBS. Serial 10-fold dilutions were cultured on BHIB agar containing gentamicin and clindamycin to determine CFU/g stool. For each experiment, the mice were divided into six experimental groups (*n* = 5–12/group). The first group (sham) was kept as the vehicle-treated control, and the second and third groups were given zerumbone (30 or 60 mg/kg/day p.o.). All groups were given antibiotics in drinking water during the experimental period. The other three groups consisted of ETBF-infected mice administrated vehicle (200 μL/20 g/day p.o.,) or zerumbone (30 or 60 mg/kg/day p.o.,) daily for 7 days, according to the experimental design (Figure 1). All materials were dissolved in a vehicle of 0.9% saline. Control groups were given the vehicle daily for 7 days as appropriate. Administration of each drug was initiated simultaneously with oral inoculation of ETBF.

### 4.4. Western Blot Analysis

HT29/C1 cells were washed twice with PBS and then lysed with radioimmunoprecipitation assay (RIPA) buffer (Life Technologies, Carlsbad, California, USA) containing a protease inhibitor cocktail (Roche, Munich, France) and phosphatase inhibitors (Roche, Munich, France). For analysis of inflammatory proteins in distal colonic tissues, distal colonic tissues (200 mg) were homogenized in 0.5 mL RIPA buffer (Life Technologies, Carlsbad, CA, USA) containing a protease inhibitor cocktail and phosphatase inhibitors. The cell lysates or distal colon lysates were incubated on ice for 20 min. The HT29/C1 cell and distal colon lysates were then centrifuged (12,000× *g*, 15 min), and the supernatants were separated. Lysates were separated by SDS-PAGEs and transferred to a nitrocellulose membrane (Life Technologies, Carlsbad, CA, USA). For measuring alterations in proteins of HT29/C1 cells or distal colon of mice, the lysates of HT29/C1 cells or distal colon were analyzed by appropriate application of primary antibodies [E-cadherin (Clone C36, BD Biosciences, San Jose, CA, USA), NF-κB p65 (Clone D14E12, Cell Signaling Technology, Danvers, MA, USA), IκB-α (Clone 44D4, Cell Signaling Technology, Danvers, MA, USA), p-IκBα (Clone 14D4, Cell Signaling Technology, Danvers, MA, USA), PARP (Clone 46D11, Cell Signaling Technology, Danvers, MA, USA) or GAPDH (Clone 6C5, Calbiochem, San Diego, CA, USA), IL-1β (ab53032, Abcam, Cambridge, UK), iNOS (Clone C-11, Santa Cruz Biotechnology, Dallas, TX, USA), COX-2 (Clone D5H5, Cell Signaling Technology, Danvers, MA, USA) and p-STAT3 (Clone D3H7, Cell Signaling Technology, Danvers, MA, USA), PCNA (Clone PC10, Santa Cruz Biotechnology, Dallas, TX, USA), Bcl-2 (Clone N-19, Santa Cruz Biotechnology, Dallas, TX, USA)] and then incubated with the appropriate horseradish peroxidase-conjugated secondary antibody [anti-rabbit IgG antibodies (Jackson ImmunoResearch, West Grove, PA, USA) or anti-mouse IgG antibodies (Jackson ImmunoResearch) for 4 h at room temperature. Immunoreactive proteins were detected using enhanced chemiluminescence of ECL kit (Bio-Rad Laboratories, San Francisco, CA, USA) and quantified by densitometry. To detect nuclear NF-κB p65, nuclear lysates from HT29/C1 cells were obtained by Nuclear/Cytosol Fractionation Kit (BioVision, Milpitas, CA, USA) according to the manufacturer′s instructions.

### 4.5. Quantitative RT-PCR (qRT-PCR)

Total RNA was isolated from whole distal colon using TRIzol reagent (Life Technologies, Carlsbad, CA, USA) according to the manufacturer’s instructions. RNA was reverse transcribed using the high-capacity cDNA synthesis kit (Invitrogen, Carlsbad, CA, USA). For each sample, two replicates were performed. Using standard amplification protocols, samples were amplified using the TaqMan gene-specific primers on a 7500 Real-Time PCR System (Applied Biosystems, Carlsbad, CA, USA). A preamplification step was performed using Toyobo Master Mix Kit (Toyobo, Osaka, Japan) and gene-specific probes used for the TaqMan PCR. Gene expression was normalized by *gapdh* expression (Thermo Fisher Scientific, Carlsbad, CA, USA). Results were expressed as 2^∆∆Ct^, and fold change was determined by comparison to the untreated control group.

### 4.6. Trypan Blue Exclusion Assay

HT29/C1 were seeded in 6-well culture plates and incubated for 48 h. The HT29/C1 were treated with different concentrations of zerumbone (6.25 to 100 μM). The NF-κB inhibitor BAY 11-7082 (10 μM) was treated as indicated. After 24 h, supernatants were removed, and cells were harvested using trypsin/EDTA (Life Technologies, Carlsbad, CA, USA) for 10 min at 37 °C. Separated HT29/C1 cells were mixed with DMEM with 10% FBS in order to neutralize trypsin. Trypan blue dye (0.4%) was added to the cell suspension to obtain a 1 to 2 dilution (example: 200 μL of cells to 200 μL of trypan blue dye), and the mixture was pipetted up and down. The percentage of viable cells was calculated by dividing the number of viable cells by the number of total cells and multiplying by 100 for % viable cells = (1.00 − (Number of blue cells ÷ Number of total cells)) × 100.

### 4.7. ELISA

Hemolysis-free sera were collected from ETBF-infected C57BL/6 mice through cardiac puncture. After coagulation, blood samples from each mouse were centrifuged at 4 °C for 12,000× *g* for 30 min, and the sera was transferred to a new microfuge tube. Sera was stored immediately at −80 °C before analysis. The concentration of murine KC (functional homologue of human IL-8) was measured by ELISA (R&D system, Minneapolis, MN, USA) according to the manufacturer instructions.

### 4.8. Nitric Oxide Assay

Nitric oxide was measured as the amount of nitrite, which is the stable end product of NO metabolism. Sera of mice were centrifuged at 4000 rpm for 30 min, and the supernatants were collected and measured by a colorimetric assay using the Griess reaction (Invitrogen, Carlsbad, CA, USA). Sera of individual mice (100 μL) were incubated with an equal volume of Griess reagent and incubated at room temperature for 10 min. After incubation, the absorbance of wells was measured at 550 nm by a microplate reader (TECAN, Mannedorf, Swiss).

### 4.9. Histologic Assessment of Colonic Inflammation

Colons from mice were washed with PBS to eliminate fecal contents and opened longitudinally along the mesenteric border and Swiss-rolled from the proximal to distal end. The Swiss-rolls were placed in 10% neutral buffered formalin for 48 h and transferred to 70% ethanol and processed to generate formalin-fixed paraffin-embedded (FFPE) tissues. FFPE blocks were sectioned by a microtome (Leica, Wetzlar, Germany) to make tissue sections (4 μm) for histology and immunohistochemical staining. Histological assessment of colon inflammation was performed in a double-blind manner using hematoxylin and eosin (HE)-stained sections. The histological scoring was performed based on severity of inflammation, extent of injury, regeneration, and crypt damage. The final inflammation score was calculated by the sum of the scores for all parameters. Colon inflammation was evaluated as follows: 0, normal; 1, mild increase in immune cells and no colonic epithelial alterations; 2, a moderate increase in immune cells and mild colonic epithelial proliferation; and 3, severe increase in immune cells and aberrant colonic epithelial proliferation with extensive loss of crypt architecture. Crypt length of colon was determined by analysis of 20 well-oriented crypts per mice using the image analysis software LAS 2.0 (Leica, Wetzlar, Germany). Inflammation score or crypt length was calculated as the median of individual measurements in two colonic sections made for each mouse.

### 4.10. Immunohistochemistry

Immunohistochemistry (IHC) was performed for analysis of macrophage distribution in distal colons of mice. Formalin-fixed paraffin-embedded sections were de-paraffinized and rehydrated using xylene and ethanol-water gradient. Murine macrophage-specific F4/80 antibody staining was performed according to standard protocol. Briefly, tissues were treated with antigen retrieval using citrate buffer (microwave boiling in antigen unmasking solution), and the primary antibody F4/80 (purchased from ATCC) was applied overnight at 4 °C, followed by biotinylated secondary antibody for 1 h at room temperature. The ABC-HRP kit was used to amplify the signal and visualization of antibody was performed using the DAB detection kit (Vector Laboratories, Burlingame, CA, USA). Slides were counterstained with hematoxylin for 10 min and mounted with glass coverslips. F4/80^+^ cells was quantified in representative 10 randomly selected 200× field per specimen. Representative F4/80^+^ cells of distal colon per mice were calculated as the median number of all number of F4/80^+^ cells in individual measurements made for each mouse. Representative images were taken using an optical microscope and rendered using Adobe Photoshop (Adobe, San Jose, CA, USA).

### 4.11. Data Analysis

We performed subsequent two-group comparisons of biological interest using the nonparametric two-tailed Mann–Whitney *U* test. All analyses were performed using GraphPad Prism 6.0 (La Jolla, San Diego, CA, USA).

## 5. Conclusions

In summary, we report that oral administration of zerumbone decreases ETBF-induced colitis in C57BL/6 mice through inhibition of NF-κB signaling. Zerumbone does not impact ETBF colonization nor BFT-induced E-cadherin cleavage. In vitro results indicate that zerumbone directly inhibits BFT-induced NF-κB activation in colon epithelial cells accompanied by down-regulation of IL-8 expression and consequently reduction of infiltrated macrophages in inflammatory colon tissue.

## Supplementary Materials

Supplementary materials can be found at https://www.mdpi.com/1422-0067/20/18/4560/s1.
