# Supplementary material for: Zerumbone Suppresses Enterotoxigenic Bacteroides fragilis Infection-Induced Colonic Inflammation through Inhibition of NF-κΒ
Source: Int J Mol Sci. 2019 Sep 14;20(18):4560. doi: 10.3390/ijms20184560 (PMC6770904; doi:10.3390/ijms20184560)
Supplement: Supplementary file 1 [file ijms-20-04560-s001.pdf]

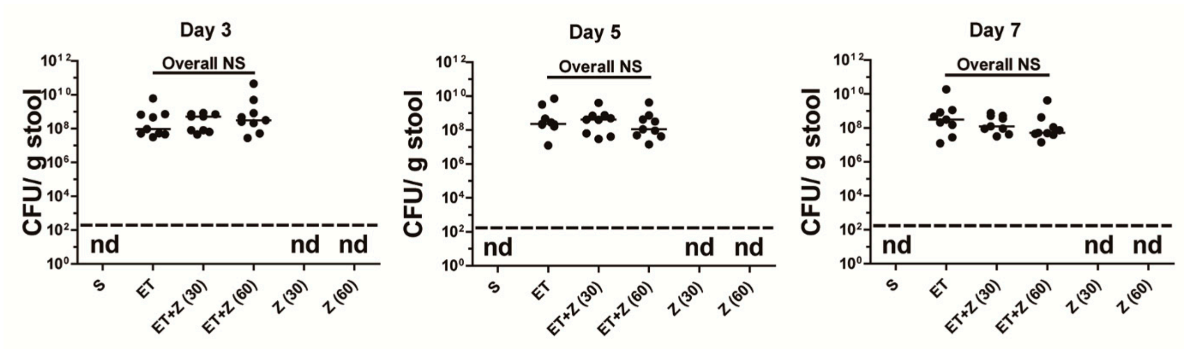

Supplementary figure S1. Zerumbone does not impact ETBF colonization in mice. WT-ETBF colonization was assessed by stool plating. S, sham; ET, ETBF; Z (30), Zerumbone (30 mg/kg); Z (60), Zerumbone (60 mg/kg); CFU, colony-forming units; nd, not detected. Scatter plot. Horizontal bar, median. ns, no statistical significance.
